# Early Flowering and Rapid Growth Become Advantageous over Non-Lodging Plants in Field Pea Under Early Drought

**DOI:** 10.3390/life16101711

**Published:** 2026-10-13

**Authors:** Baurzhan Arinov, Gulmira Khassanova, Marzhan Kuzbakova, Satyvaldy Jatayev, Makpal Kanatkyzy, Gulnar Kylyshbayeva, Crystal Sweetman, Yuri Shavrukov

**Affiliations:** 1 Institute of Agronomy and Forestry, Saken Seifullin Kazakh Agrotechnical University, Astana 010000, Kazakhstan; b.arinov@kazatu.edu.kz (B.A.); m.kuzbakova@kazatu.edu.kz (M.K.); s.jatayev@kazatu.edu.kz (S.J.); 2 Kazakh Research Institute of Agriculture and Plant Growing, Almalybak, Almaty District 040909, Kazakhstan; kanatkyzy.makpalll@mail.ru; 3 Faculty of Natural Sciences, Central Asian Innovation University, Shymkent 160000, Kazakhstan; kuntun-gulnar@portal.caiu.edu.kz; 4 College of Science and Engineering, Biological Sciences, Flinders University, Adelaide, SA 5042, Australia; crystal.sweetman@flinders.edu.au

**Keywords:** early drought, flowering time, lodging, seed yield, terminal drought

## Abstract

Field pea genotypes with tall or semi-dwarf height and with regular or semi-leafless type leaves and tendrils differ in their reactions to drought depending on plant growth and development, impacting final seed yield. In the current opinion paper, arguments are presented showing that terminal drought at the post-flowering and maturing stage can be better tolerated by semi-dwarf and semi-leafless plants with a strong vertical standing ability compared to tall and leafy pea genotypes that suffer severe lodging. On the other hand, early drought at the vegetative stage strongly reverses the preference for faster growing and early flowering tall pea plants with regular leaves. It seems that short and semi-leafless plants lose the advantage of their non-lodging ability in early drought conditions. Meanwhile, drought occurring in the middle of the flowering period causes mixed results, whereby either group of pea genotypes can produce higher seed yield depending on local adaptations. This opinion-paper highlights the importance of characterising the impacts of drought stress at various plant developmental stages, separating differential responses of plants to early and terminal drought stress for pea production. The expected timing of drought, which is specific to different regions, must be used in the development of new cultivars with improved seed yield stability.

## 1. Introduction

Lodging is the main limitation of dry pea (*Pisum sativum* L.) productivity under favourable conditions, and is due to weak stems, particularly in the basal part [1]. There are two ways to address this issue. The first option is to reduce plant height (PH) and produce semi-dwarf pea plants with a shorter stem length that have a more stable vertical growth habit and standing ability [2]. This is similar to the semi-dwarf cereals that gave rise to the ‘green revolution’ [3]. The second option is to maximise the use of tendrils, a modified leaf trait observed in the genus *Pisum* and several other legume species. Pea plants with multiple tendrils and a semi-leafless trait can have reduced lodging because the tendrils support stronger vertical growth [4,5].

Extensive breeding practice has confirmed that shorter PH and semi-leafless pea cultivars produce significantly higher seed yield in favourable conditions compared to severely lodging tall pea genotypes with regular (conventional) leaves. This is the reason that almost all current and new pea cultivars are semi-dwarf and semi-leafless worldwide [6,7,8]. However, this preference for semi-dwarf and semi-leafless over tall and leafy pea genotypes is changing dramatically in response to drought.

Drought can arise in different ways and over various time periods. The reduction in water availability may be gradual and slow in onset, or stronger and more sudden, particularly if it occurs in combination with heat. For many crop species, the timing of drought, i.e., the stage of plant development at which water limitation occurs, can determine the level of impact on growth [9,10]. However, despite the enormous importance and interest in drought for plant breeding, the effect of drought on yield in dry pea at varying growth and developmental stages with differing plant heights, leaf and tendril types remains unclear [11,12].

The aim of the current opinion paper is to analyse our knowledge in this area, focusing on the impact of the timing of drought on pea yield, and in particular how this is influenced by growth habit and lodging. All available published data were summarised and it have found that the degree of yield loss due to terminal drought at the mature plant stage was not impacted in semi-dwarf and semi-leafless compared to tall and leafy pea genotypes. Drought occurring in the middle of the flowering period caused mixed results, whereby peas exhibiting either growth phenotype could produce higher seed yield depending on specific genotypes and local factors. Most notably though, early drought at the vegetative stage was less detrimental to faster growing and early flowering tall pea plants with regular leaves. In this way, short and semi-leafless type plants lose their yield advantage in early drought conditions.

The central hypothesis of the current opinion paper included the proposed mechanism for the reaction of pea plants in response to drought. Regardless of PH (tall or semi-dwarf) and leaf type (regular or semi-leafless), pea plants must be prepared for the possibility of drought, and better prepared plants will ‘win’ and produce greater seed yield. Therefore, a combination of the following factors becomes critical: Rapid growth and development, large leaf area with effective photosynthesis and the ability to remain up-right, helping their growth. In conditions of terminal drought, especially when the early growing period has been very wet, the pea type prioritised will be the combination that produces the best non-lodging ability. Obviously, such plants would be semi-dwarf and semi-leafless, whereas intermediate place will be semi-dwarf with regular leaves, and tall and semi-leafless plants, but tall plants with regular leaves will fall to the very bottom of the yield production scale due to severe lodging. If drought occurs in early vegetative stages of plant development, however, the situation will be reversed, where priority is shifted to rapid plant growth but not for lodging ability. Only tall plants with regular leaves provide rapid growth resulting in higher seed yield compared to other pea types. Semi-dwarf and in particular semi-leafless plants have less photosynthetic area and slower plant growth resulting in the worst seed yield in the conditions of early developing drought, whereas intermediate position again will be occupied by semi-dwarf plants with regular leaves, and tall and semi-leafless plants. The presented opinion paper will cover this aim and provide arguments for the central hypothesis below.

## 2. PH and Leaf Type Are Related to Seed Yield in Pea Plants

The major tendency in pea breeding in multiple countries over recent times has been the drive for semi-dwarf and semi-leafless plants, producing cultivars with reduced lodging and higher yields. In Germany, 24 normal-leafed and 30 semi-leafless genotypes evaluated in different environments, demonstrated that semi-leafless genotypes had a 51% higher seed yield and 40% higher plant biomass overall [13]. Similar results were reported in pea genotypes grown in different countries and conditions, for example, in Africa (Ethiopia), the lodging score of tall pea cultivars had a significant negative association and direct effect on reduced seed yield [14].

Semi-leafless *afila* pea plants with more tendrils are more upright growing, with stronger connections between plants making for an entire massive non-lodging pea field [15]. Although initial introgression of *afila* alleles was accompanied by reduced seed yield (10–11%) due to decreased photosynthetic leaf area [16,17], this disadvantage was surpassed by the advantage of reduced lodging, and they are widely distributed in current pea cultivars and breeding lines, e.g., in the USA [6] and Russia [18]. In Colombia, leafless pea hybrids containing *afila* alleles showed equal seed yield to the recurrent parent by the third backcross, indicating for a successful breeding strategy using molecular-genetic analyses [19].

Despite the recorded improvement in yield with semi-leafless pea cultivars and the lower prioritisation for growth of traditional leafy cultivars in the field, there is still some relevance for more traditional leafy phenotypes [20]. For example, in organic farming, leafy genotypes can better compete with weeds, and under certain environments, some leafy genotypes can produce similar yields to semi-leafless genotypes [13].

## 3. Photosynthesis as One of the Major Mechanisms of Plant Growth Under Drought

In leafless pea genotypes, light interception by the plant canopy is lower than for leafy genotypes, however, it increases during vegetative growth and declines after mid-flowering. Furthermore, the semi-leafless plants convert intercepted light into dry biomass with higher seed yield due to their stronger non-lodging ability [21]. Such semi-leafless pea cultivars with their upright habit had cooler canopies, resulting in greater seed yield than regular leafy cultivars. Overall, the semi-leafless and up-right plant habits were superior traits that not only reduced lodging but maintained a lower canopy temperature resulting in higher seed yield [7,10]. However, in the early stages of plant development, photosynthesis is more effective in pea plants with a much bigger area of regular leaves compared to semi-leafless genotypes. Such effective photosynthesis and active metabolites stimulate rapid growth of plants with regular leaves, which gives greater opportunity for preparation in advance of the coming drought.

## 4. Terminal Drought in Post-Flowering Plants

Countries in the Mediterranean region typically experience terminal droughts in mature plants at the end of their reproductive period. Türkiye has a typical Mediterranean climate and it was reported that after a wet spring, various semi-leafless and shorter pea cultivars kept a stronger upright position and produced seed yields up to 6% higher than taller and lodging genotypes with regular leaves [22,23]. Nevertheless, it was reported that some genotypes like P104 with regular leaves also showed significantly higher seed yield, but only in drier and hotter conditions in field trials [24]. For forage biomass pea production in Türkiye, the result was different, where leaf type had no effect on forage yield in various years and locations [25,26]. Therefore, it can be concluded that in favourable conditions, seed yield was higher in semi-leafless pea genotypes whereas forage biomass has been reported as recording a similar range for pea genotypes with semi-leafless and regular leaf types in the Mediterranean climate of Türkiye. Detailed data for this and following sections are present in Supplementary Table S1.

In Italy, also with a Mediterranean-type climate, very similar results were reported about 37 pea cultivars, and it was concluded that shorter pea plants with semi-leafless phenotypes showed non-lodging upright growth with significantly higher seed yield compared to traditionally used tall and leafy genotypes that had much larger vegetative biomass [20]. These results were confirmed in the model of genomic selection for seed yield under terminal drought compared with phenotypic and marker-assisted selection [27]. Additionally, 12 commercial and registered pea cultivars were tested in field trials in three locations of Southern Italy, with a Mediterranean climate, and Central and Northern Italy with intermediate and sub-continental climates, respectively. The authors reported significantly higher seed yield in pea plants with short PH at the southern site. However, in contrast, in the central and especially in the northern site, taller pea plants had significantly higher seed yield with preferable early flowering time [28].

## 5. Mid-Season Drought Around Flowering Time

Drought and high temperatures, especially during flowering and seed formation, have a very big impact on seed yield in pea. In Germany, three leafy and four semi-leafless cultivars with similar PH were tested with dry and hot periods during the reproductive stage, from start of flowering to maturity. Vegetative biomass production was reduced, resulting in shorter plants and less lodging. In the end result, seed yield was reported as significantly higher in leafy compared to semi-leafless cultivars. The difference in seed yield was even stronger in four isogenic lines with conventional leaves compared to low-yielding semi-leafless isogenic lines grown in the same conditions under drought [29].

In Canada, experiments in two locations, Rosthern and Saskatoon, were qualified as stressed with both drought and heat during the entire flowering period. Twenty-four commercial pea cultivars, half with regular leaves and another half semi-leafless genotypes, had very similar seed yield without any difference between pea plants grown in conditions of combined drought and heat stresses, but pod abortion was increased. Only one group of leafy and vining pea plants with bright-green coloured leaves showed significant reduction of seed yield in drought and heat stress conditions, which occurred during the plant flowering period. Such combined drought and heat stresses reduced the period of flowering, resulting in lower seed yield in all pea genotypes [7,30].

## 6. Early Drought with Young Plants

Early drought can sometimes occur in various countries, especially those with a continental climate, and can range from light to very strong with corresponding mild or devastating effects. A pea crop growing in Bulgaria experienced early drought during vegetative plant growth. In total field trials, breeding line No. 29 with rapid growth and taller PH = 74 cm had higher seed yield for about 20 and 10% than shorter standard cultivars, Kerpo and Kristal (PH = 51–48 cm), respectively [31]. It can be concluded that at least one component of seed yield in this experiment was early drought and the genotype-dependent reaction of pea plants with variable PH but similar lodging ability under dry conditions.

**Figure 1:**
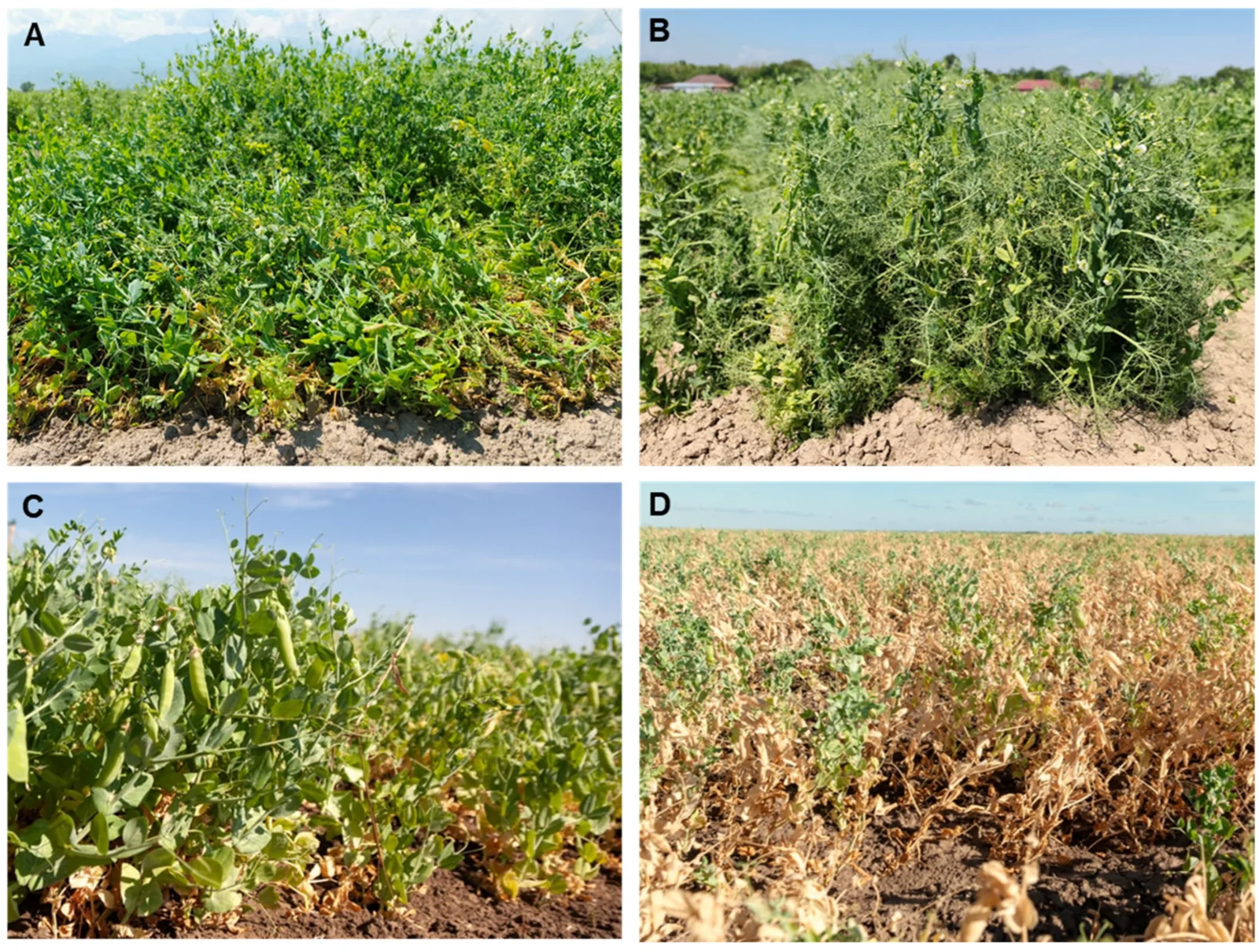
Examples of pea genotypes growing in wet and sufficient soil moisture (**A**,**B**) in comparison to the same genotypes grown in conditions of early drought (**C**,**D**). Tall plants with regular (conventional) leaves had severe lodging (**A**) and absence of lodging (**C**). Semi-dwarf and semi-leafless plants in wet conditions (**B**) and under drought (**D**). Images were made and presented by the authors in different regions and years in Kazakhstan.

In Kazakhstan, a Central Asian country with a strong continental climate, seed yield was assessed in six major pea cultivars and 60 accessions. The short and semi-leafless pea genotypes showed better results in favourable conditions with high soil moisture, whereas tall pea genotypes with regular leaf type were more productive in dry conditions. It was concluded that the lodging problem remains significant in wet conditions, whereas with severe drought, an earlier start and faster growth of taller plants with regular leaves and early flowering can become much more important traits for better drought tolerance and seed production [32]. This statement is illustrated in Figure 1.

Similarly, in the Central European regions of Russia, semi-leafless and shorter PH pea genotypes were preferable in conditions of sufficient and above-average rains. In contrast, regions with early drought required rapidly grown taller pea genotypes with conventional type leaves [18,33,34].

In an experiment with pots, supported pea plants were subjected to drought from a very early developmental stage (21 day-old plants) until maturity compared to well-watered controls. A positive correlation was reported between aboveground dry vegetative biomass and seed yield, but it was much stronger for plants under drought (*r* = 0.89) compared to controls (*r* = 0.68). This means that pea plants grew faster with taller PH in dry conditions and they produced greater vegetative biomass and seed yield than other genotypes. In contrast, plants with shorter PH developed slower and had less biomass and seed yield in the same experiment with drought [35].

Overall, to mitigate yield losses it is very important to consider the timing and forecast of drought stress and the likely stage of ontogenesis at the time of drought, when selecting pea genotypes.

## 7. Forecast Drought Can Induce Early Flowering and Rapid Plant Development

Plant reactions to drought can be very different but for the purposes of the current opinion paper, we want to mention only drought tolerance and drought escape. In general, drought tolerance means adaptation of plants to the conditions of limited water availability resulting in stomatal closure and reduction of metabolites and photosynthesis in the attempt to minimise water loss during the dry period. There are huge numbers of publications about drought tolerance, reflecting the importance of the topic [36,37]. However, drought escape is another mechanism more relevant to the subject of the current opinion paper.

In contrast to tolerance, drought escape refers to the rapid growth of plants prior to the onset of a forthcoming drought. Plants with a drought escape mechanism have intensified photosynthesis, absorb, use and evaporate water faster, resulting in a quick metabolism and rapid plant growth [38]. Drought intensity and duration define the severity of drought stress but this is unpredictable as it depends on many factors in combination, such as occurrence and distribution of rainfall, evaporative demands of the atmosphere and the moisture storing capacity of soils. However, intensity and duration of drought is outside the focus of the current opinion paper so we refer the reader to other relevant publications [39,40]. Some losses are due to intermittent drought during the vegetative phase while others are due to terminal drought during reproductive development [41].

As mentioned above, drought can often result in earlier flowering but also in reduced biomass and seed yield. Several examples of this are given below. Pea breeding line BS3 was tested in UK sites with varying levels of available water. Pea plants had a longer flowering period in one site (BP-B) with high moisture holding capacity. It was associated with high seed yield and dry biomass production. In contrast, increased drought in another site (SB) resulted primarily in reduced plant biomass but also in quicker plant development and earlier flowering plants [21].

In pea mapping populations grown in Canada, QTLs were identified in three populations of recombinant inbred lines (RIL) with highest explained percentage of phenotypic variance in multiple environments including QTLs for days to flowering (47.9%), plant height (65.1%) and seed yield (54.2%) indicating the high importance of flowering time and plant height for seed yield in pea RIL populations [42].

An inter-species hybrid with super-ultra-short vegetation was developed between garden pea, *Pisum sativum* L., and the most distant relative, *P. fulvum* Sibth. et Sm. The hybrid plants flowered just 24 days after germination providing every possible opportunity for escape of drought and any other stresses. A genetic map was established in a RIL population and marker-assisted selection proposed to be used as very early flowering pea genotypes in pea breeding programs [43,44].

In environments with expected forecast drought, selection for a shorter time to flowering could be highly successful. However, such selection for early flowering may not always increase yields in climates with unpredictable drought. In grain legumes including pea, it may also result in less capacity to respond to the additional rainfall in more favourable seasons [45].

In conditions of drought, the strategy selection of early flowering genotypes has been extensively used by legume breeders. Yield potential and early flowering in forecast drought can be a major component in legume breeding. However, progress towards earliness may be limited by the negative correlation between early flowering and yield potential. Early growth vigour, high percentage of ground cover, and high biomass were traits associated with forecast early drought resulting in higher harvest index and finally with higher seed yield. However, the success of increasing legume production in early drought largely depends on rapid plant development that enables the crop to survive in early soil-water deficits [46].

For comparison with other legumes, drought coinciding with the vegetative, flowering and pod-filling stages in pigeonpea plants under rainout shelters reduced seed yield in studied breeding lines by 15–40% in different years [47], and in chickpea ranged from 26 to 61% [48]. In lentil, *Lens culinaris* Medik, 242 lines were studied in three locations contrasting in rainfall in Northern Syria and Lebanon for lodging rate and plant biomass production. In the dry field site, there was no lodging at all because PH of lentil plants was too short (22 cm in average) and biomass production was low at about 1.5 t/ha. In contrast, in two trials with higher moisture, the biomass production was two-fold higher, exceeding 3 t/ha despite more considerable lodging of taller lentil plants (32–33 cm in average) [49]. This means that under drought there is no problem with lodging but rapidly growing plants can provide larger biomass and higher seed yield.

## 8. Conclusions

The development of field pea plants with shorter stature and semi-leafless phenotypes represented a revolutionary step for crop production due to the mitigation of lodging. This is the reason why the absolute majority of modern commercial pea cultivars are short and semi-leafless in most growing areas.

However, drought conditions can alter this priority, depending on a number of factors. One of the most important is physiological aspect with a focus on its effect on photosynthesis. Rapid growth and plant development can be supported by large leaf area with effective photosynthesis and the ability of pea plants to remain up-right, helping their growth. Furthermore, when pea crops are grown in conditions of terminal drought, especially in countries with a Mediterranean climate, there remains a clear yield benefit for semi-dwarf and semi-leafless genotypes over tall and leafy pea plants.

In contrast, in countries with a strong continental climate, early drought is much more typical during the vegetative stage, which can strongly affect growth of pea plants. In such early drought conditions, there is a shift in pea plant growth under drought and genotypes with the fastest start to the growing season are more likely to outperform slower growing pea plants, usually with reduced PH and semi-leafless type growth. Results in Kazakhstan and other similar countries, and in experiments with early drought, demonstrate this. Based on the current evidence, tall pea plants with regular leaves, that are early developing and with rapid growth, are better prepared for the forecasted early drought, while short and semi-leafless plants with slower growth and less photosynthetic area show poorer performance. These plant responses are likely influenced by genotype × environment interactions and conclusions were summarised in Table 1 and more detailed information is present in Supplementary Table S1. Therefore, the rapid growth and development, usually in tall pea plants with regular leaves, that were disadvantageous traits in wet conditions, become much more advantageous in areas subject to early drought conditions. The major physiological mechanism behind such a conclusion is photosynthesis and the capacity quick growth and development of large leaf area with effective photosynthesis prior to early forecast drought relating to drought escape of pea plants.

The agricultural conclusion from the current opinion paper is related to right prediction of possible drought correct choice of pea cultivars or their combinations taking into account that terminal drought and early stage forecast drought can have a very different impact on pea plants with various PH and type of leaves.

## Figures and Tables

**Table 1:** Impact of drought in different developmental stages on tall plants with conventional leaves and semi-dwarf and semi-leafless pea genotypes (More detailed information is present in Supplementary Table S1).

Plant Developmental Stage on Occurrence of Drought	Type of Pea Plants	
	Tall Plants with Conventional Leaves	Semi-Dwarf and Semi-Leafless Pea Genotypes
Terminal drought at the post-flowering stage	Severe lodging and strong seed loss	Best performance of seed yield due to strong upright standing ability
Mid-season drought at flowering time	Mixed results, with some genotypes showing advantages in certain environments and experiments	
Early drought on young vegetative plants	Plants developing and flowering earlier are better prepared with no lodging ability	Plants have slower growth and less photosynthetic area showing poorer performance

## Data Availability

The original data presented in this study are included in the research paper. Further inquiries can be directed to the corresponding authors.

## Supplementary Materials

The following supporting information can be downloaded at: https://www.mdpi.com/article/10.3390/life16101711/s1. Table S1: Details of studied papers with mentioned country, climate, genotypes and yield of pea cultivars, breeding lines and genotypes.
