# Supplementary material for: Early Flowering and Rapid Growth Become Advantageous over Non-Lodging Plants in Field Pea Under Early Drought
Source: Life (Basel). 2026 Oct 13;16(10):1711. doi: 10.3390/life16101711 (PMC13705981; doi:10.3390/life16101711)
Supplement: Supplementary file 1 [file life-16-01711-s001.zip › life-4601979-supplementary.pdf]

Supplementary Materials

Supplementary Table S1. Details of studied papers with mentioned country, climate, genotypes and yield of pea cultivars, breeding lines and genotypes.

Country	Genotype	Yield	Conditions	Refe-rence
**Mediterranean climate and terminal drought**				
Türkiye	Niva, Countess (semi-leafless); Solo, Maro (leafed); Mascot, Masterman (tare-leaved).	Yield was 6-20% higher in Niva and Countess compared to other cvs.	Different sowing seasons (spring and autumn) and seeding rate (25-100 seeds/m2).	[22]
	Six genotypes: Ulubatli and Kirazli (semi-leafless), and P98, P101, Golyazi and Urunlu (leafed).	Highest yield was in Ulubatli and Kirazli (2.93 and 2.90 t/ha). Lowest yield was in P101 and P98 (2.55 and 2.66 t/ha). 6% advantages of semi-leafless genotypes.	Eight diverse locations in the coastal regions with very low altitudes.	[23]
	Eight genotypes P57B, P57K (semi-leafless); P101, P104, P105, 020, P51, P98 (leafed).	Highest yield was in P57B and P57K (1.79 and 1.75 t/ha). Genotype P104 had high yield (1.55 t/ha) but in hot and dry trial.	Two locations, under dry and irrigated conditions, during two years.	[24]
Italy	Thirty-seven recent cultivars	Three top-yielding cvs., Spirale, Alliance and Attika, were semi-leafless and spring-type (3.93 t/ha for the group, n=20).	Two locations, three groups of genotypes, 11 Mediterranean, 20 spring and 6 winter.	[20]
	12 commercial and registered cultivars with various PH, in three locations and during two seasons.	In location (1), yield was 1.92-1.34 t/ha with PH 51.8-72.5 cm; in location (2), yield was 1.16-2.91 t/ha with PH 36.7-74.0 cm; and in location (3) yield was 4.19-5.52 t/ha with PH 60.5-95.8 cm.	Three locations: (1) Bari, Southern Italy, Mediterranean climate (2) Perugia, Central Italy, transition subcontinental and Mediterranean climate; (3) Lodi, Northern Italy with typical subcontinental climate.	[28]
**Mid-season drought around flowering time**				
German y	Three cvs. Belinda, Finale and Messire (normal-leafed) and four cvs. Juno,	Yield (dt/ha): 49.6 and 32./1 dt/ha in normal-leafed cvs and isogenic lines, respectively; and 46.9 and	One season for both cvs and isogenic lines. Yield was in ‘dt/ha’ units = ratio of yield	[29]

	Kasino, Solara and Terese (semi-leafless); and isogenic lines	26.3 dt/ha in semi-leafed cvs. and isogenic lines, respectively.	without wire/against wire × 100)	
Canada	12 cvs. normal leaves and 12 cvs. semi-leafless	In combined drought and heat conditions, yield in all cvs. was similar (about 250 g/m^2), excepting one group of vining plants with regular, bright-green coloured leaves (about 150 g/m^2).	Two locations, six environments	[7]
**Early drought with young plants**				
Bulgaria	Five hybrid lines and two control cvs., Kristal and Kerpo	Yield in breeding line No.29 was 3.38 t/ha, 10-20% higher than cvs., Kristal and Kerpo.	Three season trials with early drought in two seasons	[31]
Kazakhstan	Six major cvs. and 60 germplasm collection.	Yield (kg/m^2) in two seasons in location (1): Tall and mixed leaf type cvs.: 0.168-0.174 and 0.025-0.033; Short and semi-leafless cvs.: 0.177-0.182 and 0.041-0.042. Yield in location (2): Tall and mixed leaf type cvs.: 0.123-0.176 and 0.032; Short and semi-leafless cvs.: 0.076-0.086 and 0.012-0.014. Germplasm collection had similar trend.	Two seasons and in two locations: (1) Akmola region, early drought and sufficient or excessive rains after; (2) Karaganda region, early drought and either rains or stronger drought after.	[32]
Russia	Various genotypes including cvs. Nord and Orlus (semi-leafless), and Orlovchanin and Truzhenik (leafed).	Variability of yield between years was 4.8-6.4-fold in cvs. Nord and Orlus and 3.1-3.4-fold in cvs. Orlovchanin and Truzhenik.	Nine years field trials in years with favourable and drought conditions.	[33]
	Two cvs. L190-02 and Faraon (semi-lealess), and Temp and L135-03 (leafed).	Yield in cvs. L190-02 and Faraon was 2.46-3.18 t/ha; and in cvs. Temp and L135-03 was 2.93-2.96 t/ha.	Three seasons of field trials with early drought in two years.	[34]
